# Regulation of metaplasia and dysplasia in the stomach by the stromal microenvironment

**DOI:** 10.1038/s12276-024-01240-z

**Published:** 2024-06-03

**Authors:** Jared D. Rhodes, James R. Goldenring, Su-Hyung Lee

**Affiliations:** 1 Program in Cancer Biology, Nashville, TN USA; 2 Epithelial Biology Center, Vanderbilt University School of Medicine, Nashville, TN USA; 3 Section of Surgical Sciences, Nashville, TN USA; 4 Department of Cell and Developmental Biology, Nashville, TN USA; 5 Nashville VA Medical Center, Nashville, TN USA

**Keywords:** Gastric cancer, Cancer microenvironment

## Abstract

Research on the microenvironment associated with gastric carcinogenesis has focused on cancers of the stomach and often underestimates premalignant stages such as metaplasia and dysplasia. Since epithelial interactions with T cells, macrophages, and type 2 innate lymphoid cells (ILC2s) are indispensable for the formation of precancerous lesions in the stomach, understanding the cellular interactions that promote gastric precancer warrants further investigation. Although various types of immune cells have been shown to play important roles in gastric carcinogenesis, it remains unclear how stromal cells such as fibroblasts influence epithelial transformation in the stomach, especially during precancerous stages. Fibroblasts exist as distinct populations across tissues and perform different functions depending on the expression patterns of cell surface markers and secreted factors. In this review, we provide an overview of known microenvironmental components in the stroma with an emphasis on fibroblast subpopulations and their roles during carcinogenesis in tissues including breast, pancreas, and stomach. Additionally, we offer insights into potential targets of tumor-promoting fibroblasts and identify open areas of research related to fibroblast plasticity and the modulation of gastric carcinogenesis.

## Gastric pre-cancer: unveiling fibroblast roles in tumor microenvironment

This review summarizes how metaplasia (normal cells changing into different types) turns into dysplasia (abnormal cell growth) in the stomach due to injury and the role of stroma during the process. Led by Dr. James R. Goldenring, the team has discovered that the stomach develops metaplasia to heal damaged tissue and suggested several biomarkers to define the change. They recently found that certain types of metaplasia repopulate the stomach lining after damage and can progress into the next stage under the influence of the microenvironment. The current study provides insights into how the stromal components in the stomach contribute to carcinogenesis, focusing on fibroblast subpopulations. This could be important for future gastric cancer treatments.

This summary was initially drafted using artificial intelligence, then revised and fact-checked by the author.

## Introduction

### Progression of metaplasia to dysplasia

Metaplasia is a response to injury and a process by which normal mature cells of one tissue lineage are replaced by cells resembling a dissimilar and differentiated cell type observed in other tissues^1^. The metaplastic response represents the most extensive example of lineage adaptation to acute and chronic injury in the stomach. Importantly, the stomach is frequently exposed to harsh insults, and metaplasia serves to protect damaged tissue in part via mucin production. In response to damage to the stomach, two different types of metaplastic lineages repopulate the gastric mucosa: pyloric metaplasia and intestinal metaplasia^2^.

**Fig. 1:**
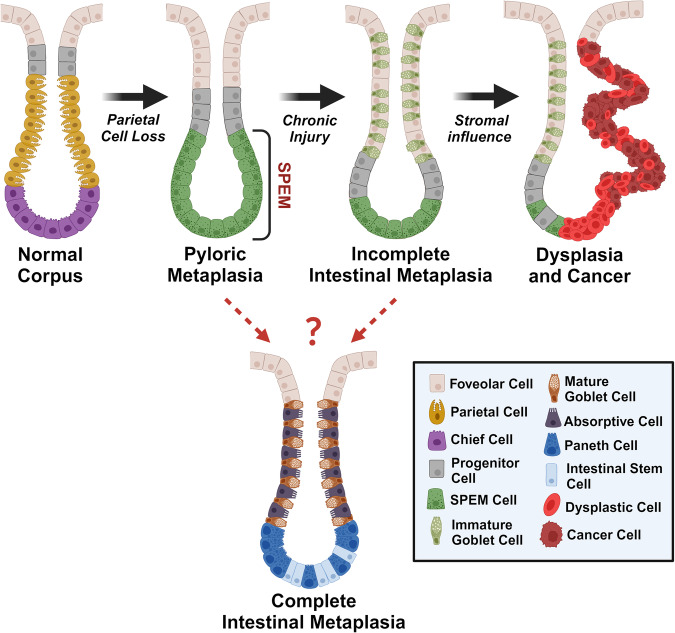
Epithelial changes during the progression of gastric carcinogenesis. The death of acid-secreting parietal cells leads to changes in cellular components and disease progression within oxyntic glands in the stomach corpus. First, pyloric metaplasia develops, which is characterized by the expansion of foveolar cells at the top of the gland and the appearance of spasmolytic polypeptide-expressing metaplasia (SPEM) cells at the gland base. Persistent inflammation during pyloric metaplasia may cause progression to incomplete intestinal metaplasia, which is characterized by the appearance of immature goblet cells with SPEM lineages at the gland bases. Pyloric and/or incomplete intestinal metaplasia may transition to complete intestinal metaplasia, which is characterized by the presence of mature goblet cells, absorptive cells, and Paneth cells. The expansion of disorganized growth in incomplete intestinal metaplasia may give rise to dysplasia, which can be promoted by stromal influences, such as fibroblasts. Dysplasia may then progress to gastric cancer. Created with Biorender.com.

Pyloric metaplasia represents the initial response to the loss of acid-secreting parietal cells through the reprogramming of digestive enzyme-secreting cells into mucus-secreting cells at the base of the gastric corpus glands (Fig. 1)^2^. The reprogramming of lineages at the base of pyloric metaplasia glands is characteristic of spasmolytic polypeptide-expressing metaplasia (SPEM), which was first identified in 1999 following investigations in which mice and humans infected with *Helicobacter felis* (*H. felis*) or *H. pylori*, respectively, that showed atypical expression of a mucinous spasmolytic polypeptide (trefoil factor 2; TFF2) at the base of damaged glands^3,4^. Common markers of SPEM cell lineages in humans and mice include CD44v9, aquaporin 5 (AQP5), and gastric intrinsic factor (GIF)/Griffonia simplicifolia lectin II (GSII) co-positivity^5^. Additionally, pyloric metaplasia exhibits an expansion of foveolar cells at the top of the gland, which is termed foveolar hyperplasia. Thus, pyloric metaplasia contains both foveolar hyperplasia toward the top of the gland and SPEM cell lineages at the bottom (Fig. 1).

Intestinal metaplasia subsequently develops in the context of chronic and unresolved injury, whereby cells with intestinal characteristics populate the gastric glands. Notably, intestinal metaplasia is commonly characterized by the appearance of MUC2-expressing goblet cells (Fig. 1)^2^. There are two distinct classifications of intestinal metaplasia: complete and incomplete intestinal metaplasia. Complete intestinal metaplasia resembles the small intestine with the presence of mature goblet cells and absorptive enterocytes throughout the glands and Paneth cells at the gland bases (Fig. 1). In contrast, incomplete intestinal metaplasia glands exhibit a hybrid mixture of gastric and intestinal-like lineages with immature goblet cells along the gland length and SPEM cells at the base of the glands (Fig. 1). Patients with incomplete intestinal metaplasia have the greatest risk of progressing to dysplasia or even gastric cancer, while patients with complete intestinal metaplasia have a lower risk and better prognosis^2,6^. Recent evidence has shown that intestinal metaplasia can develop from SPEM cells, as indicated by the presence of the intestinal metaplasia marker TFF3 in the stomach only after the emergence of SPEM^7,8^. Following incomplete intestinal metaplasia, dysplasia occurs in the stomach, which can progress to intestinal-type gastric cancers (Fig. 1)^2^.

Dysplasia refers to increased disorganization in terms of cellular morphology and glandular structure, where neoplasia, or uncontrolled cellular growth, arises following metaplasia. Specifically, in the stomach, dysplasia represents a nonmalignant or noninvasive transition in the epithelium^9^. Thus, dysplasia is the proximate step to gastric cancer. Recent studies have shown that TROP2 is upregulated during the progression from metaplastic to dysplastic phenotypes. TROP2 is absent in SPEM cells, but is upregulated in incomplete intestinal metaplasia and dysplastic stomach regions in humans and mice. TROP2 is absent from the normal stomach and is not detected in complete intestinal metaplasia. TROP2 thus represents a biomarker for the transition through incomplete intestinal metaplasia to dysplasia^10^. More recently, our group identified CEACAM5 and CEACAM6 as markers of dysplastic transition in the human stomach^11^. These studies allow for better characterization of carcinogenesis stages in the stomach based on known marker expression.

### Models for studying disease progression

Although methods to study the progression of carcinogenesis in the stomach are limited, acceptable models exist. Mouse models allow for the study of this process in the stomach through the induction of pyloric metaplasia. The highest risk for gastric cancer is observed in patients infected with *H. pylori*, as infection causes the death of parietal cells, leading to the development of metaplasia. If chronic infection and damage persist, gastric cancers can develop years following infection^12^. *H. pylori* infection in humans and gerbils^13^ promotes preneoplasia, and infection with *H. felis* in mice leads to the loss of parietal cells (oxyntic atrophy) and pyloric metaplasia development within 6 months to 1 year. Further progression to dysplasia can be observed within one year following infection in these mice^14^.

Since *H. felis* infection takes months to induce pyloric metaplasia development, our group established models to induce acute oxyntic atrophy in mice. The drugs DMP-777 and L635 induce parietal cell death followed by the induction of pyloric metaplasia^14,15^. Both of these drugs are parietal cell protonophores that induce parietal cell death, likely through acid influx into parietal cells^2,16^. One key difference between these drugs is associated inflammation. DMP-777 did not lead to a significant influx of immune cells, while L635-treated mice exhibited marked inflammatory infiltration. The likely reason for this is that DMP-777 is also an inhibitor of neutrophil elastase. It also takes 10–14 days for pyloric metaplasia development in response to DMP-777, whereas it only takes three days in response to L635^14,17^. Subsequently, high doses of tamoxifen were shown to induce death in parietal cells in the stomach and promote pyloric metaplasia in mice through a parietal cell-directed protonophore mechanism^16,18^. These models of acute oxyntic atrophy have been crucial in determining the steps required for the transdifferentiation of chief cells into SPEM cell lineages.

A critical step in understanding the process of pyloric metaplasia induction was the development of Mist1-Kras mice, which exhibited inducible expression of active Kras (G12D) in chief cells^14^. These mice sequentially developed pyloric metaplasia at one month after induction, followed by incomplete intestinal metaplasia at 2–3 months and dysplasia at 4 months. The origin of SPEM lineages from chief cells was later confirmed in GIF-Kras mice^19^. To understand in greater detail the progression of metaplasia and dysplasia, Min et al. generated mouse gastroid lines from Mist1-Kras mice to recapitulate the in vivo models^20^. These gastroids mimicked the characteristics of Mist1-Kras model mice and led to insights into the identification of dysplastic stem cells^20^. Importantly, macrophage infiltration within the precancerous mucosa continuously increased throughout the progression of carcinogenesis. This finding implies that some cell types within the gastric microenvironment can facilitate the carcinogenic cascade.

## Microenvironment in gastric carcinogenesis

**Fig. 2:**
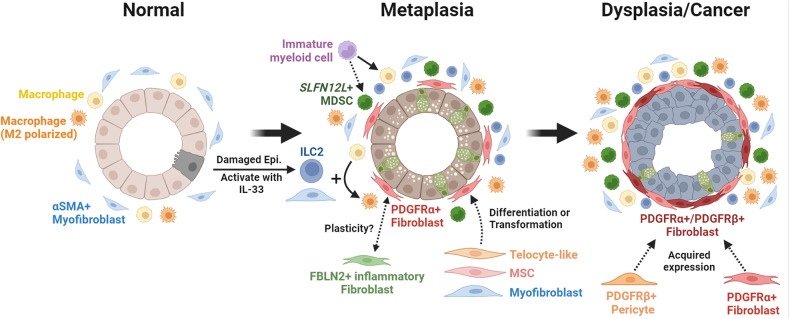
Alterations in the microenvironment during the progression of gastric carcinogenesis. Normal corpus glands in the stomach are mainly surrounded by a few myofibroblasts and tissue-resident inflammatory cells, including type 2 innate lymphoid cells (ILC2s) and polarized and unpolarized macrophages. Epithelial damage, such as parietal cell loss, initiates an immune response through the release of a variety of cytokines and chemokines. Surface cell-derived interleukin-33 (IL-33) activates ILC2s to express IL-13, leading to macrophage polarization toward the M2 phenotype and macrophage infiltration. Myofibroblasts expressing αSMA in the stomach might be involved in macrophage polarization, similar to those in prostate or pancreatic cancers. Additionally, the recruitment of myeloid-derived suppressor cells, which are characterized by the expression of Schlafen family member 12 like (SLFN12L) in humans and SLFN4 in mice, may be required for the development of metaplasia. During metaplasia development, PDGFRα+ fibroblasts expand and reach close proximity to damaged gastric glands, leading to the progression to dysplasia. Fibroblasts can originate from telocyte-like fibroblasts, mesenchymal stem cells (MSCs), or tissue-resident myofibroblasts. Fibulin-2 (FBLN2)-expressing inflammatory fibroblasts, which are associated with mononuclear immune cell infiltration, can transform into PDGFRα+ fibroblasts. Dysplastic or cancerous epithelial compartments are tightly surrounded by an increased number of PDGFRα+ fibroblasts, and some of these cells coexpress PDGFRβ. Both PDGFRα+ fibroblasts and PDGFRβ+ pericytes can be the origins of coexpressing cells. Created with Biorender.com.

The gastric microenvironment is composed of nonepithelial cells in proximity to stomach glands (Fig. 2). Most studies have focused on characterizing the microenvironment in gastric cancer tissues rather than the normal or metaplastic precancerous mucosa. However, some studies have characterized the normal stomach stromal cell composition. Single-cell RNA sequencing (scRNA-seq) of stomach tissues obtained from patients with normal stomachs or nonmalignant chronic gastritis revealed a heterogeneous population of immune and stromal cells and dynamic changes in cell composition during the progression to cancer. The cell types included B cells, T cells, endothelial cells, macrophages, and fibroblasts. Among these cell types, gastric cancer patients most notably had increased proportions of macrophages and T cells compared to those in normal tissue^21^. Additional sequencing studies have shown that gastric cancers are associated with a higher degree of immune and stromal cells, such as T cells, monocytes, dendritic cells, and fibroblasts compared to normal gastric tissue^22,23^.

The role of most inflammatory microenvironmental elements in the progression of metaplasia to dysplasia/cancer remains unclear due to the absence of appropriate experimental models and functional studies. While many of these components remain poorly understood, our group and others have identified how some inflammatory components regulate the induction and progression of pyloric metaplasia in the context of acute or chronic epithelial injury, which is specifically induced by drug treatment or *Helicobacter* infection. Previous studies have characterized the immune landscape in association with *Helicobacter* infection in mice and revealed that mononuclear leukocytes chronically infiltrate the infected mucosa and can form lymphoid follicles composed of B and/or CD4 + T cells. T and B cells, neutrophils, and macrophages are most prevalent in the mucosa following infection^24^. L635 treatment-induced acute parietal cell loss resulted in the infiltration of immune cells, including macrophages, T and B cells, and polymorphonuclear cells^14^.

The role of T cells in the development of metaplasia in the stomach has been well characterized. For example, T-cell-derived interferon gamma (IFNɣ) promotes oxyntic atrophy directly or in a B-cell-mediated manner^25,26^. Studies using severe combined immune-deficient (SCID) and Rag1 knockout mice (lacking T and B cells) have shown that the adaptive immune system is essential for parietal cell death. For example, the Th1 immune response, which is mainly driven by IFNɣ, is essential for *Helicobacter*-associated gastric pathology, but B cells may not significantly affect epithelial and stromal changes during infection^27,28^. To evaluate which adaptive immune cell type is responsible for oxyntic atrophy, researchers used microMT mice (lacking B cells) and found that these mice lost parietal cells normally compared to immunocompetent mice. In comparison, T-cell deficient (TCRβδ^-/-^; TCR double knockout) mice infected with *H. felis* did not develop oxyntic atrophy^28^. These results indicated that T cells, but not B cells, were necessary to induce death in parietal cells in the mouse stomach. In addition, further studies have demonstrated that populations of myeloid-derived suppressor cells (MDSCs) are required for the induction of parietal cell death by *Helicobacter* infection (Fig. 2)^29^. Therefore, it is likely that an interaction between MDSCs and T cells is important for the initiation of *Helicobacter*-induced parietal cell loss. L635-induced acute oxyntic atrophy bypasses the mechanisms that are required for T-cell-mediated *Helicobacter*-induced parietal cell loss and therefore permits the study of the function of T cells in SPEM following parietal cell death^30^. Further work by our group revealed that T cells were not required for the progression of pyloric metaplasia, especially in L635-treated mice^30^. Similar to wild-type L635-treated mice, Rag1- or IFNɣ-knockout mice developed advanced proliferative SPEM. Thus, T-cell activity is required for the induction of parietal cell loss but not for the induction of SPEM.

Macrophages are important players in the microenvironment because they engulf pathogens, serve as professional antigen-presenting cells, and mediate a wide array of immune responses^31^. Our group showed that macrophages were necessary for the induction and progression of SPEM in L635-treated mice using clodronate liposomes to deplete mucosal macrophages (Fig. 2)^30^. These macrophages derived from L635-treated mice, as well as macrophages derived from human patients with intestinal metaplasia, manifested primarily M2 polarization (Fig. 2)^30^. Compared with M1 macrophages, M2 macrophages exert anti-inflammatory effects and are involved in promoting tumorigenesis^32^. Although this work implicated M2 macrophages in metaplastic progression, the identification of additional mediators is required for a more complete explanation of the microenvironmental influences of SPEM.

Further work by our group and others demonstrated that type 2 innate lymphoid cells (ILC2s) were necessary for pyloric metaplasia development (Fig. 2)^30,33–35^. Specifically, ILC2 activation in the gastric mucosa requires interleukin-33 (IL-33) released from the damaged epithelium (Fig. 2). IL-33 and IL-13 drive macrophage polarization to the M2 phenotype (Fig. 2), which is necessary for the induction of SPEM. Therefore, our group proposed that IL-33 stimulates ILC2s to release IL-13, which promotes the induction of SPEM following parietal cell death (Fig. 2)^34,35^. These results were confirmed in an adrenalectomy model of oxyntic atrophy in mice^33^.

Fibroblasts have been less characterized in the context of gastric carcinogenesis, although several papers examined human gastric cancer samples and suggested that fibroblasts were indispensable for tumor invasion and metastasis^36–38^. Normal fibroblasts are involved in extracellular matrix deposition, wound healing, and migration. In cancer, fibroblasts become polarized and further promote tumorigenesis as cancer-associated fibroblasts (CAFs). CAFs can promote tumorigenesis by secreting soluble factors such as cytokines or by altering the extracellular matrix^39^. However, while the term CAF may suggest a particular population of cells, it does not indicate which specific populations of fibroblasts are responsible for promoting carcinogenesis. Studies in the stomach have indicated that fibroblast populations identified in cancer-bearing tissues are also present in the normal, noncancerous mucosa (Fig. 2)^11,36^. Therefore, we will next examine how populations of fibroblasts can change in the context of pathological conditions in the stomach and in other organs.

## The origins of fibroblasts and their subsets during the progression of gastric carcinogenesis

Fibroblasts are generally found throughout the body and develop from connective tissue-creating mesenchyme following the establishment of the embryonic mesoderm germ layer^40,41^. Here, we will focus on the origins of fibroblast populations in the precancerous and cancerous gastric mucosa.

**Fig. 3:**
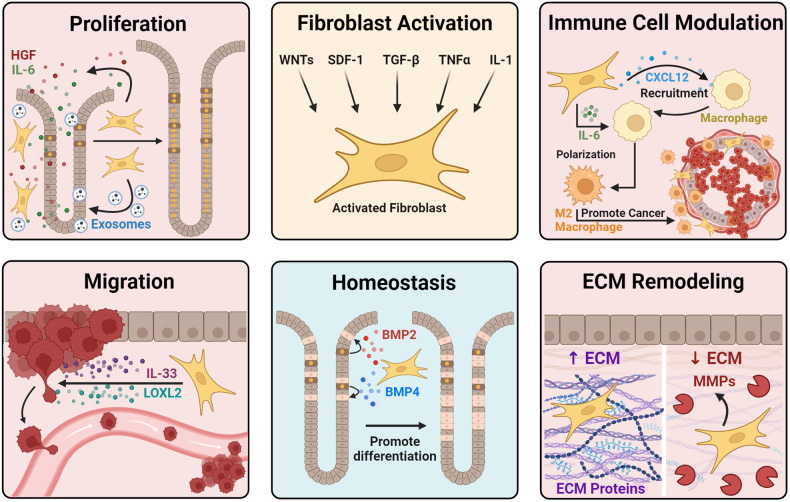
Functions of activated fibroblasts during carcinogenesis. Resting fibroblasts can be activated by several signaling molecules, including TGF-β and WNT ligands. Following activation, fibroblasts can promote carcinogenesis through multiple mechanisms. Activated fibroblasts promote the proliferation of epithelial cells through growth factor and cytokine release. Additionally, activated fibroblasts promote the migration of cancer cells through the secretion of IL-33 and LOXL2. Activated fibroblasts are also involved in the remodeling of the extracellular matrix (ECM) by depositing ECM proteins such as collagen and laminin or breaking down the ECM via matrix metalloproteases (MMPs). During carcinogenesis, immune cells such as macrophages can be recruited by fibroblast-derived chemokines such as CXCL12. After recruitment, IL-6 released by activated fibroblasts promotes the polarization of macrophages to a protumorigenic M2 state. Other types of fibroblasts also play antitumorigenic roles by promoting the terminal differentiation of epithelial cells through the release of bone morphogenic protein (BMP) to maintain tissue homeostasis. Created with Biorender.com.

Bone marrow-derived mesenchymal stem cells (MSCs) can serve as precursors for fibroblasts in the mucosa, especially in the context of mucosal injury (Fig. 2)^42,43^. One group cultured MSCs with the phosphoprotein osteopontin, which upregulated transforming growth factor beta (TGF-β), leading to a CAF phenotype (Fig. 3)^44^. Similarly, Quante et al. reported that MSCs could differentiate into myofibroblast populations following TGF-β stimulation in mouse models of metaplasia and dysplasia^45^.

Fibroblasts in normal or cancerous tissues are functionally and phenotypically heterogeneous populations^46^. Therefore, the classification of these distinct populations is necessary to precisely understand their mechanisms of action in various cancer types. For many years, the dogma has been that fibroblasts are mainly protumorigenic in nature (Fig. 3). Therefore, it was assumed that depletion of fibroblasts would suppress tumorigenesis. However, it has been demonstrated that the ablation of total fibroblasts can favor cancer cells in models of pancreatic ductal adenocarcinoma (PDAC)^47,48^. These studies provide further evidence of the heterogeneity of fibroblast subsets in tumorigenesis and suggest that some fibroblasts are tumor suppressive (Fig. 3). There has been a lack of studies on gastric-specific fibroblast subsets; however, subsets are better defined in other organ types. Therefore, we will first examine general population definitions across systems prior to focusing on the stomach.

We will first focus our attention on myofibroblasts, since they represent a widely accepted form of fibroblasts associated with cancers. Myofibroblasts were first identified in experiments in which injured rats exhibited modified fibroblasts distinct from those commonly observed. These altered fibroblasts were characterized by increased fibrils (similar to smooth muscle cells), irregularly shaped nuclei, and increased extracellular components attached to their cell membranes^49^. Furthermore, these fibroblasts exhibited prominent expression of the *ACTA2* gene, which encodes alpha-smooth muscle actin (αSMA). These morphological and transcriptional characteristics have defined myofibroblasts as contractile cells that promote wound healing^50^. Myofibroblasts exhibit an increased propensity to remodel the extracellular matrix (ECM) and have been implicated in tumor promotion in several cancer types, such as pancreatic and breast cancers^51,52^. Myofibroblasts can expand following activation by signaling molecules such as TGF-β, WNT ligands, tumor necrosis factor-alpha (TNFα), and stromal cell-derived factor 1 (SDF-1) (Fig. 3)^40,53^. Additionally, there is evidence that myofibroblasts can develop from myeloid cells, endothelial cells, and adipocytes^54–56^. Inflammatory CAFs (iCAFs) are also common and can differentiate from pancreatic stellate cells after being cultured with IL-1^57^. Inflammatory fibroblasts maintain the inflammatory milieu in tissues by expressing inflammatory signaling molecules such as IL-6^58,59^.

Recent scRNA-seq-based studies have more precisely identified CAFs and their molecular and functional heterogeneity in pancreatic and breast cancers. Multiple studies on CAFs in PDAC have defined the CAF subgroups iCAFs and myofibroblasts (myCAFs), which affect the composition of the microenvironment, in humans and mice^47,51,60,61^. Elyada et al. reported that iCAFs expressed *PDGFRA*, *CXCL12, CXCL2, HAS1*, and *IL-6*, and myCAFs highly expressed *ACTA2*. Among several papers on CAFs, only Elyada et al. briefly mentioned *FBLN2* as an iCAF marker in PDAC^61^. Fibulin-2 (FBLN2) is involved in establishing the ECM through interactions with laminins, fibronectins, and collagens^62^. Intriguingly, antigen-presenting CAFs (apCAFs), which can present antigens to T cells via MHCII expression, has also been identified in PDAC^61^.

Similarly, fibroblast subsets have been well-defined in breast cancers. One study defined four populations of fibroblasts in breast cancers by using flow cytometry. CAF-Subset 1 (CAF-S1) and CAF-S4 cells were positive for αSMA, indicating a myofibroblast phenotype. CAF-S1 cells were distinct from those in subset 4 based on their expression of fibroblast activation protein (FAP). CAF-S3 and S4 cells were enriched in fibroblast-specific protein 1 (FSP1) and PDGFRβ, and subset 4 also expressed αSMA. CAF-S2 cells were negative for all markers, including CD29, FAP, FSP1, αSMA, PDGFRβ, and CAV1^63^. Further work by this group showed that these subsets were maintained in the metastatic lymph nodes of breast cancer patients^64^. Interestingly, additional findings from these studies revealed *FBLN2* expression only in CAF-S1 cells. Other groups have defined fibroblast populations similar to those in PDAC: myCAFs (which express αSMA) and iCAFs (which express Ly6c1 and HAS1)^61,65^. apCAFs have been described in breast cancer based on their expression of MHC II, similar to that in PDAC^65^. Interestingly, in breast and pancreatic cancers, myCAFs have been implicated as the most protumorigenic subset^56^.

Compared to pancreatic and breast tissues, there is a limited understanding of definitions of fibroblast populations in the stomach. Notably, it was not until recently that multiple groups began addressing this issue. Using single-cell analysis of gastric cancer tissues from human patients, Kumar et al. identified a novel subpopulation of fibroblasts that was positive for *FAP* and *INHBA* and was positively associated with poor patient prognosis^66^.

By comparing regions of gastric cancer and adjacent normal tissue, Li et al. observed the presence of four distinct populations of fibroblasts in the stomach: myCAFs, iCAFs, pericytes, and extracellular matrix CAFs (eCAFs). Among them, iCAFs in the stomach were similar to iCAFs observed in the pancreas, through the expression of IL-6 and CXCL12. Notably, this group discovered a previously unrecognized subset of fibroblasts termed eCAFs with high expression of *POSTN*, a gene important for promoting the migration of epithelial cells. Interestingly, these eCAFs were observed primarily in the distal stroma and were associated with an overall decrease in the survival of patients. Additionally, eCAFs were shown to associate with M2 macrophages^36^.

Finally, Kim et al. described three populations of CAFs in gastric cancers. Similar to the classification of cells in PDAC, iCAFs were characterized by the upregulation of cytokine, chemokine, and matrix metalloproteinase-encoding genes, and myCAFs were characterized by the expression of various markers, such as *POSTN*, *TPM1*, and *TAGLN*. iCAFs were observed prominently in cancerous samples compared to normal and intestinal metaplasia samples, and myCAFs were not significantly distributed in one lesion. Finally, this work defined a new subset known as intermediate CAFs (inCAFs) that expressed *PDGFRA* and *POSTN*^22^.

Although these studies provide insights into fibroblast subsets during gastric malignancy, they do not address earlier stages of the disease. Our group recently demonstrated the presence of four distinct subpopulations of fibroblasts in the normal, metaplastic, and cancer-bearing human gastric mucosa through scRNA-seq^11^. First, telocyte-like *PDGFRA*+ fibroblasts (FBS1; fibroblast subset 1) were more abundant in metaplastic and cancerous tissues than in adjacent normal tissue (Fig. 2). Inflammatory *FBLN2*+ fibroblasts (FBS2) were associated with inflammatory cell infiltration or lymphoid follicles. *ACTA2* + /*PDGFRB-* myofibroblasts (FBS3) were most abundant in adjacent normal and cancerous tissues. Finally, pericyte-like *ACTA2* + /*PDGFRB*+ fibroblasts (FBS4) were observed in adjacent normal tissue and were expanded in proximity to some cancers (Fig. 2). These studies demonstrated that distinct populations of fibroblasts were more prominently expanded in association with metaplastic or dysplastic mucosal lesions (Fig. 2)^11^.

Interestingly, isolating fibroblasts from different pathologic lesions had distinctly different effects on the behavior of metaplastic gastroids in 3D and 2D air–liquid interface (ALI) cocultures^11^. Compared with inflamed normal-derived fibroblasts, 3D coculture of cancer-derived or metaplasia-derived fibroblasts with metaplastic organoids increased proliferation and dysplastic transition. This phenotype was recapitulated when an ALI 2D culture technique was used to plate and grow metaplastic gastroids directly on top of monolayers of fibroblasts. Metaplastic gastroids that were cocultured with metaplasia- or cancer-derived fibroblasts showed decreases in the metaplastic markers AQP5 and CD44v9 and increases in the dysplastic markers TROP2 and CEACAM5^11^. Furthermore, conditioned media from cancer-derived and metaplasia-derived fibroblasts enhanced dysplastic transition but not the proliferation of metaplastic-derived organoids. These findings indicate that stable secreted factors are released from the metaplasia- or cancer-derived fibroblast populations to promote this dysplastic phenotype and that other potentially unstable factors might be necessary to specifically enhance growth.

Recently, Nowicki-Osuch et al. found multiple populations of fibroblasts in precancerous metaplastic human esophagus tissue and stomachs^67^. Specifically, two subsets of these lesions were characterized by the expression of *PDGFRA* and *ACTA2*, as determined by scRNA-seq. These subsets were most abundant in association with esophageal intestinal metaplasia, gastric intestinal metaplasia, and SPEM. Notably, *PDGFRA+* fibroblasts expressed genes encoding secreted proteins that are important for the ECM, such as POSTN and COL6A1, as well as chemokines, such as CXCL14. In contrast, myofibroblasts expressed *ACTA2* and *ACTG2*, which are important for cell motility. These gene signatures indicate an important role for these fibroblasts in migrating and remodeling the ECM. These fibroblasts seem to be important in the development of metaplastic phenotypes, suggesting that there are similarities between stromal compartments in precancerous gastric and esophageal lesions that may establish a protumorigenic environment. More work must be done to characterize whether these fibroblast subsets can directly promote tumorigenesis.

## Mode of action of fibroblasts in carcinogenic progression

We will first focus on fibroblast-derived secreted factors, since they have been well characterized as factors that regulate the epithelium. Multiple groups have reported that fibroblast-derived IL-6 promotes the proliferation of gastric cancer cells (Fig. 3) via STAT3 phosphorylation^68,69^. Additionally, gastric cancer cell migration can be regulated by lysyl oxidase-like 2 (LOXL2), which is secreted by fibroblasts^70^. One study revealed that hepatocyte growth factor (HGF) secreted from fibroblasts was important for inducing CAF activation in surrounding normal fibroblasts and promoting the growth of gastric cancer cells (Fig. 3). Inhibiting HGF specifically in gastric CAFs decreased overall tumor progression^71^. Furthermore, IL-33 derived from CAFs can bind to its receptor, ST2, on gastric cancer cells to promote migratory and invasive phenotypes (Fig. 3). Depletion of IL-33 in CAFs decreased metastatic tumors in nude mice^72^. These functional studies suggest that proteins secreted by fibroblasts are essential for supporting cancer progression.

Interestingly, CAFs have been shown to modulate the immune microenvironment to promote cancer development. Multiple studies on prostate, colorectal, and pancreatic cancer have shown that CAFs secrete factors such as CXCL12 and IL-6 that increase M2 macrophage recruitment and polarization (Fig. 3), resulting in increased growth and migration of cancer cells^73–75^. Although these studies have not been recapitulated in the stomach, we know that M2 macrophages promote the development of metaplasia in the stomach (Fig. 2)^30^. Therefore, it seems likely that CAFs in the stomach can contribute to the recruitment and polarization of macrophages to an immunosuppressive state that enhances carcinogenesis. Additionally, CXCL12 produced by CAFs promoted the invasive phenotype of gastric cancer cells in tissue culture^76^. This finding suggested that secreted chemokines such as CXCL12 could act directly on the gastric epithelium to enhance tumorigenesis and modulate immune responses. Furthermore, in vitro and in vivo findings in the colon, pancreas, and lung suggest that CAFs express programmed cell death ligand 1 (PDL1), which can bind to PD1 on T cells to suppress their activity and promote tumor properties^77–79^.

Another method by which CAFs can promote tumorigenesis involves the secretion of exosomes (Fig. 3). Richards et al. reported that exposure of PDAC-derived CAFs to chemotherapy greatly increased exosome release^80^. These exosomes contained mRNAs of the transcription factor SNAIL, which enhanced cancer cell proliferation and survival^80^. In the stomach, exosomes can promote the aggressive behavior of cancer cells. The transcription factor HSF1 promotes the expression of inhibin subunit beta A (INHBA) and thrombospondin 1/2 (THBS1/2) in CAFs. Once synthesized, these proteins are packaged into exosomes and secreted to promote cancer progression^81^. Furthermore, one study revealed that extracellular vesicles containing Annexin A6 derived from CAFs could promote resistance to pharmacological treatment in gastric cancer cells^82^. These findings indicate that extracellular vesicles and exosomes produced by CAFs can promote tumorigenesis.

Although there is a lack of functional data related to fibroblasts in precancerous lesions in the stomach, we can look at other organ systems to make inferences about their role in the gut. The transcription factor ETS2, which is expressed in αSMA+ fibroblasts, was increased during pancreatic acinar-ductal metaplasia (ADM) events^83^. Knockout of *ETS-2* in mouse pancreatic fibroblasts suppressed ADM development and reduced pancreatic epithelial proliferation. Interestingly, *ETS-2* KO fibroblasts exhibited significantly less binding of ETS2 to the promoters of cytokines and chemokines involved in immune cell recruitment. These data suggest that fibroblasts affect the production of chemokines and cytokines that allow for immune invasion. Other studies have implicated ETS1, which is another member of the ETS family, in the progression of cancer. In stromal fibroblasts, ETS1 is upregulated in conjunction with target genes encoding the secreted matrix metalloproteases MMP-1 and MMP-9 in colorectal and preinvasive or invasive breast cancers, highlighting the ability of activated fibroblasts to remodel the ECM (Fig. 3)^84,85^. These findings further support the role of proteins secreted from fibroblasts in promoting carcinogenesis in the precancerous milieu.

Multiple subtypes of fibroblasts promote carcinogenesis; however, some of them act conversely and regulate tissue homeostasis through bone morphogenic protein (BMP) signaling (Fig. 3). BMP receptor depletion in the gastrointestinal mesenchyme can result in the development of gastric polyps and intestinal and pyloric metaplasia^86^. Furthermore, mice expressing the BMP inhibitor noggin in the stomach showed signs of metaplasia/dysplasia development and proliferation^87,88^. BMP2 seems to be crucial for maintaining gastric mucosal homeostasis and differentiation (Fig. 3), which can be disrupted during *H. pylori* infection^89^. Therefore, restoring BMP signaling in patients with metaplastic or cancerous stomachs may be an intervention strategy. A recent study revealed that the CD105-negative fibroblast subpopulation could suppress pancreatic carcinogenesis and regulate the adaptive immune system^90^. Accordingly, understanding how fibroblast populations are involved in a carcinogenic cascade is necessary for identifying targetable mechanisms for prevention and treatment.

## How to prevent or block tumor-favorable fibroblast subsets

Although several meta-analyses have presented conflicting data about intestinal metaplasia, *H. pylori* eradication seems to cause the regression of precancerous metaplasia in a significant number of patients, indicating that metaplasia might be the last point at which a return to normal is possible^91,92^. Therefore, suppressing the progression of dysplastic metaplasia is an important therapeutic strategy. Our data have suggested that fibroblasts in metaplasia or dysplasia facilitate the cancerous progression of noncancerous metaplastic cells, and targeting fibroblasts in preneoplastic lesions may inhibit malignant transformation. However, as discussed previously, some fibroblast types exert tumor suppressive effects, as indicated by several studies showing that the ablation of CAFs unexpectedly resulted in more aggressive tumor phenotypes^47,48,90^. Therefore, we will focus on targeting protumor fibroblast subsets to promote an antitumor microenvironment. It is important to understand how targeting protumor fibroblasts may also prove to be effective in precancerous conditions.

The development of vaccines targeting CAFs has been attempted in mouse models of colon and breast cancer. Using a DNA vaccine to target FAP on CAFs increased T-cell activity and the killing of CAFs, thereby decreasing the tumor burden^93^. Additionally, targeting FAP with CAR-T cells effectively reduced the progression of lung and pancreatic cancers in mice^94^. One group showed that FAP+ CAFs in mouse models of PDAC could be depleted by conjugating a FAP antibody to a photosensitizing dye and chelator using targeted photodynamic therapy^95^. These results indicate that targeting FAP may be an effective means of treating various cancers.

Strategies targeting fibroblast-derived factors have shown promising results in suppressing malignant transformation. Recently, it was found that WNT2 secreted by CAFs in mice with esophageal squamous cell carcinoma or colorectal cancer decreased dendritic cell differentiation and antitumor T-cell activity^96^. Interestingly, this effect was reversed by treating the mice with a combination of immunotherapy and an anti-WNT2 antibody compared with immunotherapy alone, suggesting that CAF-derived WNT2 was a suitable target for combination therapy. Protein secretion is a major means by which CAFs modulate the microenvironment. Thus, targeting these proteins before they can exert their effects may be beneficial for reducing malignant progression.

Stromal reprogramming represents another promising strategy against CAFs. For example, PDGFRα/β blockade in fibrotic gastric tumors has shown promising results when combined with anti-PD-1 immunotherapy^97^. Dual treatment significantly reduced αSMA+ fibroblasts, increased immune cell populations, and slowed tumor growth in mice. These results suggest that blocking fibroblast receptor-mediated signaling in CAFs may reverse their protumorigenic functions and restore stromal elements in the tumor microenvironment. Furthermore, it was recently determined that administering vitamin D receptor ligands to pancreatic cancer mouse models reverted pancreatic stellate cells to a quiescent state that mediated an effective chemotherapeutic response^98^.

## Challenges related to gastric epithelial and stromal interactions

As stated previously, our group recently showed that conditioned media from metaplastic or cancer-derived gastric fibroblasts promoted the dysplastic transition in metaplastic gastroids. Therefore, secreted factors in the medium were sufficient to induce a change in phenotype. Sequencing revealed that cancer- and metaplastic-derived fibroblasts expressed higher levels of genes encoding secreted proteins, specifically in *PDGFRA* + FBS1 and *FBLN*2 + FBS2, compared to those in inflamed normal tissue. Notably, many of these genes were enriched in pathways involved in cellular proliferation, ECM organization, and cellular adhesion^11^. These findings indicate that factors secreted from fibroblasts are important drivers of the carcinogenic cascade. Although this work highlights the importance of fibroblasts in promoting dysplastic transition within the stomach, many mechanistic questions remain unanswered.

First, how the physical interactions of fibroblasts with the gastric epithelium can influence disease progression is poorly understood. Due to the lack of proliferation in gastroids cultured with fibroblast-derived conditioned media^11^, physical interaction or close proximity may be necessary to enhance the proliferation of the gastric epithelium. Further experiments must be conducted to examine the possible physical interactions between fibroblasts and the epithelium that are required to enhance dysplastic progression and overall cellular proliferation. It is also feasible that unstable secreted factors, such as prostaglandins or prostacyclins, in fibroblast-derived conditioned medium could stimulate proliferation. In this context, we may not observe proliferation in conditioned media experiments because these factors are labile.

Second, it remains unclear which epithelial lineages are responsible for the dysplastic transition. Our recent studies have shown that human gastroids with features of SPEM lineages, including the expression of CD44v9 and AQP5, undergo a dysplastic transition when cocultured with metaplastic- or cancer-derived fibroblasts^11^. However, the actual origin of these SPEM lineages is not clear. SPEM lineages exist in both pyloric metaplasia glands and incomplete intestinal metaplasia glands (Fig. 1)^5^. Given the isolation of these gastroid lines from regions adjacent to cancers in patient resections, they may derive from SPEM lineages at the bases of incomplete intestinal metaplasia. This question of origin remains unclear. Furthermore, we cannot rule out the possibility that dysplasia also arises from intestinalized lineages within incomplete intestinal metaplasia and that the influence of fibroblasts on these transitions is not discernable at this time but is likely important.

Finally, the plasticity and interconversion of fibroblast subsets in tissues are poorly understood. In the pancreas, 3D cultured iCAFs converted to myCAFs in 2D culture^51^. These findings suggest that fibroblast populations are dynamic and reversible, although the underlying mechanisms remain unclear. Whether these fibroblast states are dictated by the fibroblasts themselves, epithelial cells, or other cells in the microenvironment is an open area of research. Our single-cell sequencing results suggested a connection between *PDGFRA*+ telocyte-like fibroblasts and *FBLN*2+ inflammatory fibroblasts rather than between *PDGFRA*+ telocyte-like fibroblasts and myofibroblasts^11^. Tissue-specific patterns of fibroblast population plasticity may define the assembly of the procarcinogenic milieu. Furthermore, plasticity among fibroblast populations may define the geography of microenvironment interactions with epithelial lineages, and alterations in fibroblast expression of key regulators may be a critical factor in carcinogenesis. The upregulation of FAP and INHBA in gastric cancers rather than precancerous lesions is a strong indicator of the upregulation of specific mediators in fibroblasts, especially in association with frank cancers^66^. Therefore, future studies will be needed to evaluate fibroblast plasticity within the stomach and in similar tissues. Understanding fibroblast plasticity could be useful for identifying potential targets to convert tumor-promoting fibroblasts into an antitumorigenic phenotype. These gaps must be addressed to better understand the role of the stromal milieu in maintaining or dysregulating healthy gastric epithelium.
